# Effects of melatonin administration on embryo implantation and offspring growth in mice under different schedules of photoperiodic exposure

**DOI:** 10.1186/s12958-017-0297-7

**Published:** 2017-10-02

**Authors:** Lu Zhang, Zhenzhen Zhang, Feng Wang, Xiuzhi Tian, Pengyun Ji, Guoshi Liu

**Affiliations:** 1 State Key Laboratory of Animal Nutrition, Key Laboratory of Animal Genetics and Breeding of the Ministry of Agriculture, National Engineering Laboratory for Animal Breeding, College of Animal Science and Technology, China Agricultural University, Beijing, 100193 China; 2 Present Address: Department of Animal Sciences, Purdue University, West Lafayette, IN 47907 USA

**Keywords:** Melatonin, Embryo implantation, MT1, MT2, p53

## Abstract

**Background:**

Embryo implantation is crucial for animal reproduction. Unsuccessful embryo implantation leads to pregnancy failure, especially in human-assisted conception. Environmental factors have a profound impact on embryo implantation. Because people are being exposed to more light at night, the influence of long-term light exposure on embryo implantation should be explored.

**Methods:**

The effects of long photoperiodic exposure and melatonin on embryo implantation and offspring growth were examined. Long photoperiodic exposure (18:6 h light:dark) was selected to resemble light pollution. Melatonin (10^−2^, 10^−3^, 10^−4^, 10^−5^ M) was added to the drinking water of mice starting at Day 1 (vaginal plugs) until delivery.

**Results:**

Melatonin treatment (10^−4^,10^−5^ M) significantly increased litter sizes compared to untreated controls (12.9 ± 0.40 and 12.2 ± 1.01 vs. 11.5 ± 0.43; *P* < 0.05). The most effective concentration of melatonin (10^−4^ M) was selected for further investigation. No remarkable differences were found between melatonin-treated mice and controls in terms of the pups’ birth weights, weaning survival rates, and weaning weights. Long photoperiodic exposure significantly reduced the number of implantation sites in treated mice compared to controls (light/dark, 12/12 h), and melatonin rescued this negative effect. Mechanistic studies revealed that melatonin enhanced the serum 17β-estradiol (E_2_) levels in the pregnant mice and upregulated the expression of the receptors MT1 and MT2 and p53 in uterine tissue. All of these factors may contribute to the beneficial effects of melatonin on embryo implantation in mice.

**Conclusion:**

Melatonin treatment was associated with beneficial effects in pregnant mice, especially those subjected to long photoperiodic exposure. This was achieved by enhanced embryo implantation. At the molecular level, melatonin administration probably increases the E_2_ level during pregnancy and upregulates p53 expression by activating MT1/2 in the uterus. All of the changes may improve the microenvironment of the uterus and, thus, the outcomes of pregnancy.

## Background

Implantation is a crucial process that involves an intricate interaction between the embryo and uterus. The defects that occur before, during or immediately after implantation are responsible for early pregnancy loss in animals, including humans. For successful implantation, an embryo needs to be developed to the blastocyst phase and the uterus must be in a receptive state, which allows the blastocyst to communicate with the luminal epithelium [1]. Similar to other physiological processes, implantation is regulated by various factors, including hormones, cytokines, associated gene expression and protein synthesis [1, 2]. Progesterone (P_4_) and E_2_ are master regulators of implantation in both mice and humans because P_4_ and E_2_ modify the receptive status of the uterus and promote embryo implantation [3–5]. In mice, E_2_ and P_4_ perform their functions via their receptors, and mice that lack estrogen receptors (ERα) or progesterone receptor (PR-A) are infertile [6–8]. In addition, leukaemia inhibitory factor (LIF) and p53 are key factors for embryo implantation [9]. LIF acts as a downstream target for estrogen and is involved in the decidualization of the maternal endometrium for blastocyst implantation [9–11]. Additionally, p53 regulates both the basal and inducible transcription of LIF to regulate the process of implantation [9, 12, 13].

Melatonin may have a potent impact on embryo implantation. As an amphiphilic molecule, melatonin enters the cell with ease to exert its biological functions, including the regulation of the biological clock and immune responses, the detoxification of free radicals and the modification of hormone and growth factor secretion [14–17]. Melatonin also plays an important role in reproductive activities by affecting the secretion of gonadotropin in the hypothalamus via the hypothalamus-pituitary-gonads (HPG) axis to regulate sexual maturation, seasonal oestrus, reproductive behaviour, redox homeostasis and gamete protection [17, 18]. Melatonin exerts some of its functions via its receptors, whereas others are independent of receptors [19]. The differential expression of the melatonin receptors MT1 and MT2 in pregnant and non-pregnant human uteri has been reported and influences a 24-h rhythm of myometrial contractility [20]. Melatonin receptors also exist in human granulosa cells (GCs), and melatonin treatment was shown to enhance the effects of human chorionic gonadotropin-stimulated progesterone production in vitro [21–23]. In addition, melatonin treatment in the range of 10 pM-100 nM significantly upregulated the gene expression of the LH receptor [23]. The effects of melatonin on P_4_ and E_2_ production are rather complicated. For example, in growing and luteinized GCs, short-term melatonin incubation (within 48 h) results in reduced P secretion; however, long-term incubation increases P_4_ production [22, 23]. In pinealectomized rats, the embryo implantation rates and serum P_4_ levels are decreased, but with daily melatonin injection, the serum P_4_ levels are normalized [24, 25]. In pinealectomized rats, low melatonin levels lead to a reduction in P_4_ and its receptors but result in an elevation in E_2_ levels [26]. In the uteri of non-pregnant pinealectomized rats, melatonin increases the progesterone receptor (PR) levels but reduces E_2_ receptor (ER) expression [27]. In another study, melatonin significantly reduces LH and 17β-estradiol levels in plasma and downregulates the expression of ERβ and PRβ; however, it upregulates MT1 expression [28]. Melatonin might also decrease the E_2_ levels during the perimenopausal period [29].

The beneficial effects of melatonin on embryo implantation have been reported in mice and can be attributed to the regulation of hormone secretion and hormone receptor activation [30, 31]. In addition, melatonin can modulate the activity of p53 by inducing the p38-dependent phosphorylation of p53 in MCF-7 cells [32]. The activation of p53-dependent DNA damage response is mediated by MT1 and MT2 in mice [33]. These actions of melatonin could also positively affect embryo implantation. Recently, it was reported that the expression of the enzyme *AANAT,* which catalyses the synthesis of melatonin, is gradually increased during early gestation and that melatonin injection increase the number of embryo implantation sites and upregulates the expression of *ErbB1* and *p53* [34]*.*

However, it is unclear whether melatonin has other effects on offspring development and growth, particularly in animals that are subjected to long photoperiodic exposure and have truncated melatonin production with circadian disturbances. This is specifically important for modern humans [35, 36], who are being exposed to more manufactured light at night [37]. The reproductive consequences of long photoperiodic exposure in mice or humans have not yet been explored. Therefore, in the current study, we evaluated litter size, birth weight, weaning survival rates and weaning weight after melatonin treatment. We also detected the number of implantation sites in the uteri of mice that were either treated with melatonin or subjected to long photoperiodic exposure. The potential signalling pathway of melatonin in the regulation of embryo implantation was also investigated by determining the protein levels of melatonin receptors, p53 and LIF.

## Methods

### Chemicals

E_2_ and P_4_ hormone radioactive assay kits were purchased from ICN (ICN Biomedicals, Inc., Costa Mesa, CA, USA), the PVDF membrane and ECL were from Luminex xMAP - EMD Millipore (MA, USA), goat anti-rabbit IgG (H + L), goat anti-mouse IgG (H + L), HRP and rabbit anti-goat IgG (H + L) were from Jackson Immunoresearch Laboratories (PA, USA); protein extraction RIPA was from Thermo Fisher Scientific (MA, USA); the MT1 antibody (SC-390328; Santa Cruz Bio Inc., Santa Cruz, CA, USA), MT2 antibody (SC-13177; Santa Cruz Bio Inc.) and p53 antibody (SC-47698; Santa Cruz Bio Inc.) were from Santa Cruz Biotechnology; the protein marker was from Thermo Fisher Scientific; and the separation gel buffer, stacking gel buffer, 20% Tween 20 and protein sample buffer were from Bio-Rad Lab (CA, USA). Melatonin and other reagents, unless specified, were purchased from Sigma Chemical Co. (MO, USA).

### Animals

Kun Ming (KM) mice (China Experimental Animal Center of Military Medical Sciences, Beijing, China) aged 8–12 weeks were kept in a temperature-controlled room at 20 ~ 22 °C under a 12:12 light/dark cycle (lights on at 06:00 and off at 18:00). After a week of acclimation, spontaneous oestrus female mice were carefully selected and kept with sexually mature males overnight. On the following morning, females were checked for vaginal plugs. Those with vaginal plugs were selected for the subsequent experiments.

### Implantation site quantification

On days 5.5 and 7.5 of pregnancy, mice received a peritoneal injection of 2% pentobarbital sodium (0.15 ml/each) and were then injected with Chicago Sky Blue 6B via the caudal vein (0.10 ml/each). Three minutes later, mice were sacrificed by cervical dislocation. The uteri were collected, cleaned and placed on white paper. The implantation sites were counted and recorded.

### 17β-estradiol and progesterone assays

The levels of P and E_2_ were detected by radioimmunoassays (RIAs). On days 4 and 4.5 of pregnancy, mice received a peritoneal injection of 2% pentobarbital sodium (0.15 ml/each), and the blood was collected and stored at 4°C for 2 h and centrifuged for 10 min at 3000 r/min. The serum was collected and stored at −80°C for future E_2_ and P detection according to the kit’s instructions.

### Western blotting

Uteri from 4.5 day pregnant mice were collected and washed three times with PVA-DPBS, lysed in sample buffer containing 62.5 mM Tris-HCl (pH 6.7), 5% 2-mercaptoethanol, 2% sodium dodecyl sulfate, 10% glycerol, and 0.002% bromophenol blue, denatured by heating to 100 °C for 5 min and frozen at −80°C until use. The proteins were subjected to SDS-PAGE using a 10% polyacrylamide gel and then transferred to a PVDF membrane for 2.5 h under a 300-mA electric current. After blocking the nonspecific binding sites by overnight incubation in Tris-buffered saline (25 mM Tris and 150 mM NaCl, pH 7.6) containing 5% nonfat milk and 0.2% Tween 20, the membranes were incubated with the primary antibodies (dilution 1: 1000) for 2 h at 37 °C. The membranes were stripped with a buffer containing 100 mM 2-mercaptoethanol, 2% SDS, and 62.5 mM Tris–HCl (pH 6.7), then re-probed with a mouse monoclonal antibody (dilution 1:1000) directed against β-actin (Ab6276; Abcam, Inc., Burlingame, CA, USA) to confirm equivalent protein loading. The bound antibodies were detected by incubation with a secondary antibody (dilution 1:1000) (Peroxidase AffiniPure Rabbit Anti-Goat IgG (H + L)). The images were scanned in an Image Quant LAS 4000 mini luminescent image analyser (GE Healthcare Bio-Sciences, PA, USA). The protein levels were evaluated by densitometry using the Quantity One software (v. 4.52; Bio-Rad Lab).

### Experimental designs

#### Experiment I

Females with vaginal plugs (the initiation of pregnancy, day 1) were divided into five groups (10/group) and treated with different concentrations of melatonin (10^−2^, 10^−3^, 10^−4^, 10^−5^ and 0 M) in their drinking water. The water was replaced every day until delivery. After delivery, the litter size, litter weight, average pup birth weight, weaning body weight and postnatal survival rate were recorded.

#### Experiment II

Mice with vaginal plugs were divided into four groups: mice without treatment served as a control group; mice treated with melatonin (10^−4^ M) comprised the MT group; mice exposed to a long photoperiod (18 h/6 h light/dark) constituted the LE group; mice exposed to a long photoperiod (18 h/6 h light/dark) plus melatonin (10^−4^ M) treatment were placed in the LE + MT group. On Days 5.5 and 7.5 of pregnancy, mice were sacrificed and their uteri were collected for implantation site analysis.

#### Experiment III

Mice with vaginal plugs were selected and treated with or without melatonin (10^−4^ M) under the same management condition. On Days 4 and 4.5, blood was collected for the detection of serum E_2_ and P_4_ following the instructions of the assay kits. On Day 4.5, uteri were also collected to analyse the protein levels of MT1, MT2, p53 and LIF via western blotting.

#### Statistical analyses

All data were expressed as the mean ± S.E.M. ANOVA was used to analyse the normality of the data, followed by Dunnett’s post hoc test to determine the significant differences between the groups using the SPSS 18.0 statistical software (SPSS Inc., IL, USA). The significance level was set as *P* < 0.05.

## Results

### Experiment I: Effects of melatonin on pregnant mice

**Fig. 1:**
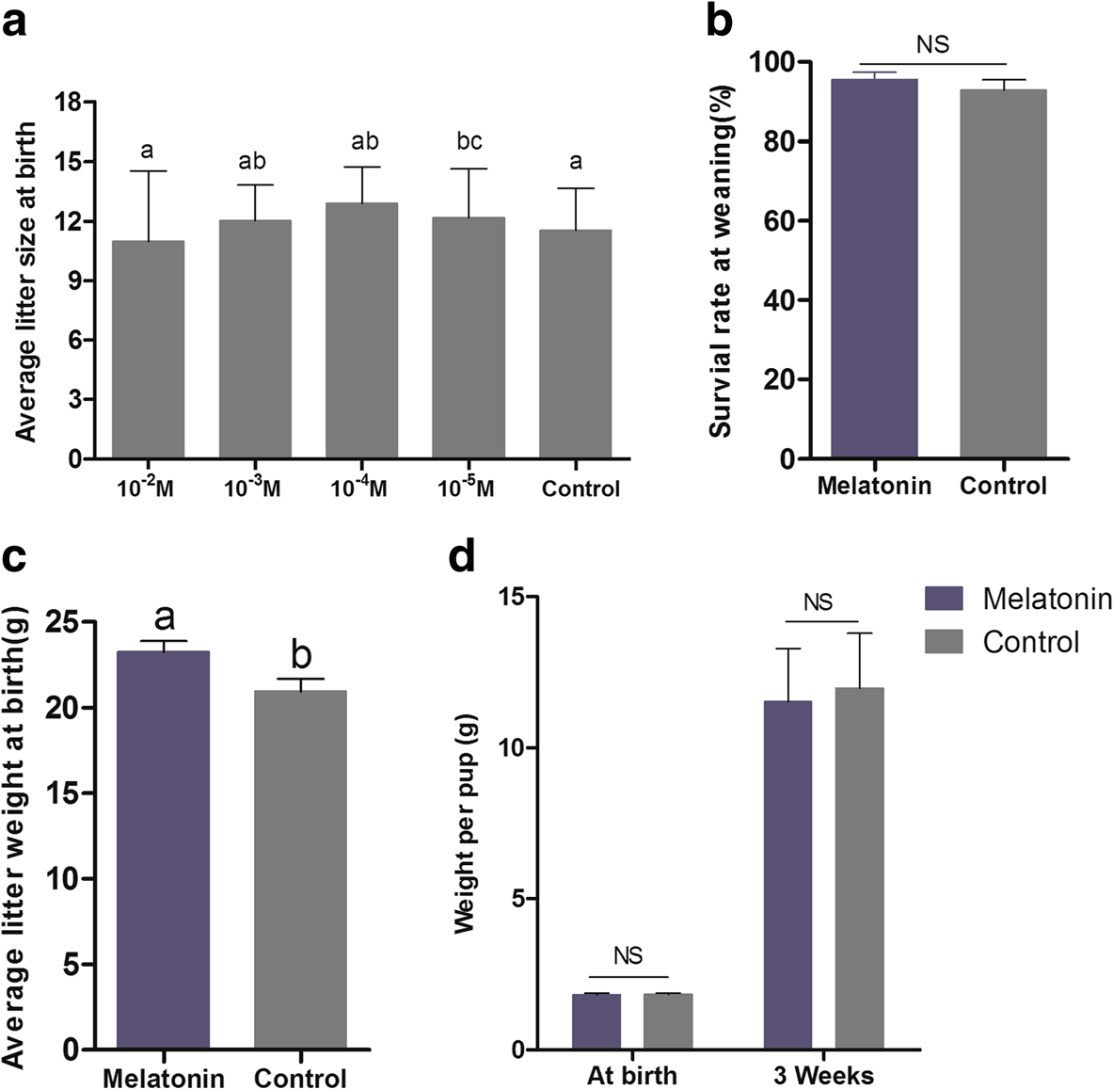
Effects of different concentrations of melatonin on offspring development and growth in mice. **a** The average litter sizes for 10^−2^, 10^−3^, 10^−4^ and 10^−5^ M melatonin-treated groups and the control group (10.97 ± 1.453, 12.00 ± 0.92, 12.9 ± 0.40 and 12.2 ± 1.01 vs. 11.5 ± 0.43, respectively). **b** The survival rates of pups at weaning in the melatonin (10^−4^ M) and control group (95.6 ± 1.78% vs. control 92.9 ± 2.62%, respectively; P˃0.05). **c** The average litter weight at birth in the melatonin (10^−4^ M) and control group (23.24 ± 0.65 vs. control 20.95 ± 0.72, respectively; *P* < 0.05). **d** The birth weight and 3rd week weaning weight per pup in the melatonin treated group (10^−4^ M) and the control group (1.8 ± 0.20 g vs. control 1.8 ± 0.17 g; 11.5 ± 0.43 g vs. control 12.0 ± 0.41 g, respectively; *P*>0.05). Data are expressed as the means ± SEM (*N* = 10 litters/group). Different superscript letters indicate statistically significant differences (*P* < 0.05)

The average litter sizes in melatonin 10^−4^ M and 10^−5^ M treated groups were significantly greater than those in the control group (12.9 ± 0.40 and 12.2 ± 1.01 vs. 11.5 ± 0.43; Fig. 1a; P < 0.05). This index was not significantly different between mice treated with melatonin at 10^−3^ M (12.00 ± 0.92; Fig. 1a) and control mice (P˃0.05). Melatonin treatment at the concentration of 10^−2^ M (10.97 ± 1.453) slightly decreased the average litter sizes of mice; however, this decrease was not significantly compared to controls (*P*˃0.05). The results indicated that the most effective melatonin concentration for litter size increase is 10^−4^ M. Thus, this concentration was selected for further studies. The survival rate of pups at the time of weaning in the melatonin-treated group slightly increased compared to the control group; however, this increase was not significantly different (95.6 ± 1.78% vs. 92.9 ± 2.62%; Fig. 1b; *P*˃ 0.05). The average litter weight at birth in melatonin (10^−4^ M) treated mice was significantly greater than control (23.2 ± 0.65 g vs. 20.9 ± 0.72 g; Fig. 1c; *P*˃ 0.05).The pups’ birth weights and 3rd week weaning weights were similar between the 10^−4^ M melatonin and control group (1.8 ± 0.20 g vs. 1.8 ± 0.17 g; 11.5 ± 0.43 g vs. 12.0 ± 0.41 g; Fig. 1d; *P*˃0.05).

### Experiment II: Effects of melatonin on embryo implantation sites on day 5.5 and 7.5 of pregnancy under different photoperiod period

**Fig. 2:**
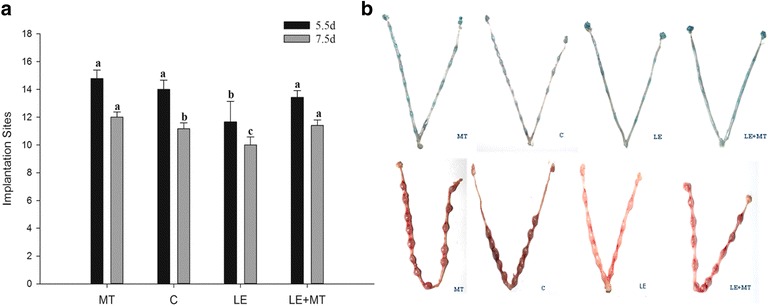
The implantation sites on Days 5.5 and 7.5 of pregnancy. MT: melatonin group; C: control group; LE: light extended group; LE + MT: light extended group treated with melatonin (10^−4^ M). **a** Implantation sites on Day 5.5 (14. 8 ± 0.62, 14.0 ± 0.67, 11.2 ± 0.43 and 13.4 ± 0.48 in the MT, C, LE and LE + MT groups, respectively) and on Day 7.5 (12.0 ± 0.37, 11.7 ± 1.48, 10.0 ± 0.58 and 11.4 ± 0.40 in the MT, C, LE and LE + MT groups, respectively). **b** Representative images of uterine implantation sites on Day 5.5 (up) and Day 7.5 (down) of pregnancy. Data are expressed as the means ± SEM (*N* = 9). Different superscript letters indicate statistically significant differences (*P* < 0.05)

The implantation sites analysis showed that there was no significant difference between the control and melatonin-treated groups at Day 5.5 of pregnancy (14.8 ± 0.62 vs. 14.0 ± 0.67; Fig. 2a; *P* > 0.05). The numbers of implantation sites in the LE group (11.2 ± 0.43) was significantly lower compared to controls (14.8 ± 0.62) and this decrease almost recovered to the control level in mice with LE plus melatonin treatment (LE + MT group) (13.4 ± 0.48) (Fig. 2a; *P* < 0.05). On day 7.5, the beneficial effects of melatonin on implantation sites were observed in the MT and LE + MT groups compared to the control and LE groups (12.0 ± 0.37 and 11.4 ± 0.40 vs. 11.7 ± 1.48 and 10.0 ± 0.58, respectively. Fig. 2a; P < 0.05). Representative images of the implantation sites in the uterus of mice with different treatments are shown in Fig. 2b.

### Experiment III

1. Effects of melatonin on serum E_2_ and P_4_ levels on Days 4 and 4.5 of pregnancy

**Fig. 3:**
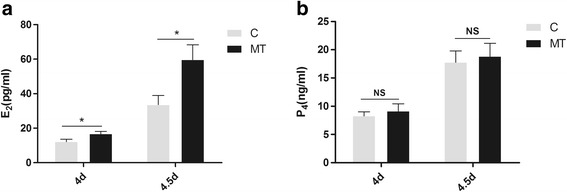
Serum P_4_ and E_2_ contents on Days 4 and 4.5 of pregnancy. The levels of P_4_ and E_2_ were determined. **a** Serum E_2_ content showed significant differences on both day 4 (59.68 ± 8.59 pg/ml vs. control 16.71 ± 1.48 pg/ml; *P* < 0.05) and day 4.5 (33.62 ± 5.34 pg/ml vs. control 12.16 ± 1.49 pg/ml; *P* < 0.5). Data are expressed as the means ± SEM (*N* = 9) Different superscript letters indicate statistically significant differences (*P* < 0.05). **b** No significant difference was found in serum P_4_ content between the control group and melatonin group on day 4 (9.12 ± 1.31 ng/ml vs. control 8.27 ± 0.73 ng/ml; *P* > 0.05) and day 4.5 (18.82 ± 2.31 ng/ml vs. control 17.76 ± 2.05 ng/ml; *P* > 0.05)

There was no significant difference in serum P_4_ content between control and melatonin treated mice on Days 4 and 4.5 of pregnancy (Fig. 3a). In contrast, melatonin treatment significantly increased serum E_2_ content on Day 4 (16.7 ± 1.48 vs. 12.2 ± 1.49 pg/ml) and on Day 4.5 (59.7 ± 8.59 vs. 33.6 ± 5.34 pg/ml) compared to the controls, respectively (Fig. 3b).

### 2. Effects of melatonin on protein levels of MT1, MT2, p53 and LIF in uteri of mice on day 4.5

**Fig. 4:**
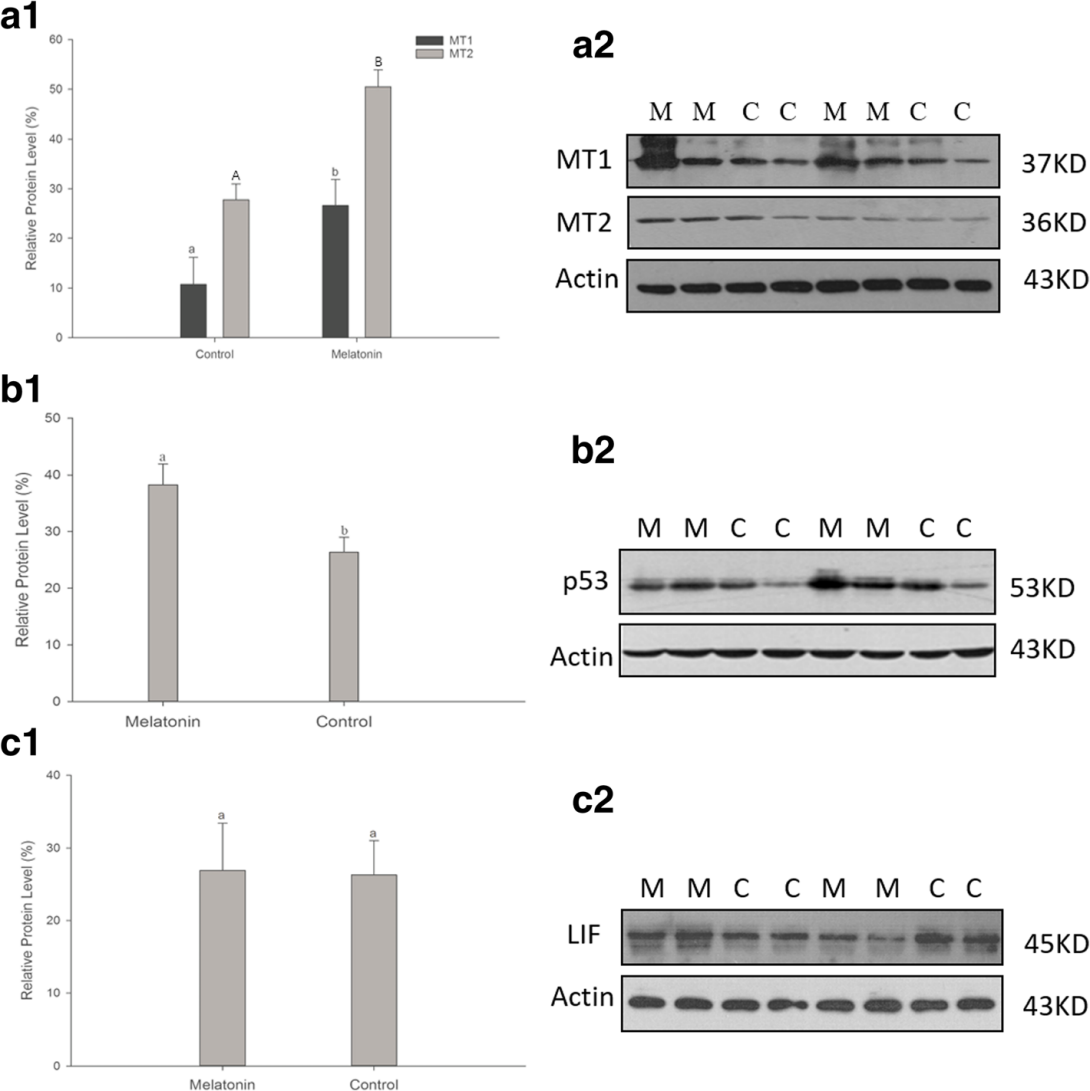
Effects of melatonin on the protein expression of MT1, MT2 and p53 on Day 4.5 in uterine tissue. (a1) On Day 4.5, MT1 and MT2 receptor expression in the uterus was significantly higher in melatonin (10^−4^ M)-treated samples than in control samples (MT1: 27.8 ± 3.23% vs. control 10.7 ± 5.38% and MT2: 50.5 ± 3.41% vs. control 26.6 ± 5.29%; *P* < 0.05), and MT2 expression was higher than MT1 expression. (b1) Uterine p53 expression significantly increased in melatonin-treated group (38.3 ± 3.69% vs. control 26.4 ± 2.65%; P < 0.05). (c1) The relative protein expression level of LIF. There was no significant difference between the melatonin and control group (26.9 ± 6.50% vs. control 26.3 ± 4.73%; *P* > 0.05). (a2, b2 & c2) Western blotting results of uterine MT1, MT2, p53 and LIF protein expression. Different superscript letters indicate statistically significant differences (*P* < 0.05). Data are expressed as the means ± SEM (*N* = 8). Different superscript letters indicate statistically significant differences (*P* < 0.05)

The signal pathway analyses demonstrated that the relative protein levels of MT1 and MT2 receptors in uteri of mice that received melatonin treatment were significantly upregulated compared to the controls, and the magnitude of increase in MT2 expression was significantly higher than that of MT1 (MT1: 27.8 ± 3.23 vs. control 10.7 ± 5.38% and MT2: 50.5 ± 3.41 vs. control 26.6 ± 5.29%; *P* < 0.05; Fig. 4a). p53 expression was also significantly higher in melatonin treated mice than in controls (38.3 ± 3.69 vs. 26.4 ± 2.65%; P < 0.05, Fig. 4b). There was no difference in the relative level of LIF between the two groups (26.9 ± 6.50 vs. 26.3 ± 4.73%; *P* > 0.05; Fig. 4c).

## Discussion

During pregnancy, the early loss of embryos is a primary factor that affects litter size in mammals. In the current study, we confirmed that melatonin treatment in pregnant mice significantly increased litter size. This observation was consistent with previous publications on mice and rats [24, 34]. Rats with a melatonin deficiency caused by pinealectomy had heavier uteri, a thicker endometrium, lower glandular organ weights, higher gland cavities, more uterine epithelial cells and increased variation capacity in attaching to the placenta; melatonin treatment reversed these alterations [24, 25]. In addition, pinealectomy in mice influenced the serum progesterone and 17β-estradiol levels, the expression of the endometrial progesterone receptor and ovarian corpus luteum numbers, and melatonin supplementation effectively reversed all these changes [25]. Some reports indicated that pinealectomy resulted in keratinization, continued oestrus and ovulation obstacles in rats [38]. Rodents with pinealectomy or under continuous illumination tended to show precocious puberty, ovarian atrophy, chronic and persistent oestrus and hyperprolactinemia [39]. These observations suggest that the melatonin circadian rhythm is essential for regulating reproductive hormones (17β-estradiol, progesterone) and for embryo development and implantation. In the current study, we observed, for the first time, that mice under prolonged light exposure had fewer embryo implantation sites. Prolonged light exposure decreases melatonin production in organisms [40, 41]. This finding indicated that the decreased embryo implantation sites resulted from melatonin deficiency. Indeed, melatonin supplementation in drinking water (10^−4^ M) counteracted the negative effects of prolonged light exposure on pregnant mice and increased their embryo implantation sites back to the control levels. P_4_ is essential for embryo implantation and pregnancy maintenance in all mammals, and E_2_ has different effects depending on the species and physiological conditions, as mentioned previously [1]. Both of these hormones are regulated by melatonin via the HPG axis. In the current study, the level of P_4_ in the blood wasn’t changed by melatonin treatment; however, melatonin significantly increased the level of E_2_. These observations were not consistent with the results obtained in rats [24] and this may be a species difference. The serum E_2_ peak is essential for uterine receptivity. A high level of E_2_ is beneficial for the implantation of an embryo, but the duration of uterine receptivity could become short under a high level of E_2_ [3, 4]. The current study showed that melatonin at 10^−5^ to 10^−4^ M increased the number of embryo implantation sites in pregnant mice. During the time window of uterine receptivity, exogenous melatonin enhanced the level of serum E_2_. Based on the results, we concluded that the increased litter size was probably achieved by the promotion of mouse embryo implantation sites induced by exogenous melatonin treatment.

Among the pregnancy outcomes, birth weight is one of the most important indexes. We observed that melatonin treatment did not affect the pups’ birth weights, weaning survival rates, or weaning weights. In addition, the average litter weight was improved in rats exposed to low-nutrient conditions with melatonin treatment [42]. The authors concluded that melatonin influenced the diastolic function of the blood vessels to enhance the efficiency of the placental nutrition supply [42]. At the same time, the microenvironment of the uterus was improved by melatonin treatment, which promoted the expression of peroxidase and antioxidant enzymes such as Mn-SOD in the placenta [42]. In another study, mice treated with melatonin via injection exhibited increased gene expression of *ErbB1, PRA, p53* and *MT2* [34]. At least it can be concluded that the administration of melatonin does not cause negative effects on offspring.

The activities of melatonin might be mediated by its receptors, since melatonin receptors are present in the ovary and uterine mesangial matrix of rats and mice [25, 41, 42]. MT1 and MT2 melatonin receptors were differentially expressed in pregnant and non-pregnant human uterine tissue, and affected the circadian rhythms of both uterine contraction and childbirth [20, 43]. To explore the signal pathway of melatonin in regulating embryo implantation, the protein levels of MT1, MT2 p53 and LIF in uterine tissue were evaluated, and MT1 and MT2 were already expressed in the uteri of mice at day 4.5 of pregnancy. In addition, exogenous melatonin administration significantly promoted the expression of MT1 and MT2. Melatonin had more profound effects on MT2 than on MT1, indicating that MT2 may be the dominant receptor to mediate melatonin’s effect on reproduction. Melatonin was shown to upregulate the expression of p53 and p21 by affecting p38 activity and increasing the phosphorylation level of p53 [32, 33, 44, 45]. A specific inhibitor of p38 MAPK (PD98059) could block the effect of melatonin on p53 [45]. The cell selective gene silencing of melatonin receptors or the use of chemical inhibitors such as luzindole could suppress the readjustment of p53 [44]. Thus, MT1 and MT2 activation by melatonin regulates the expression of p38, resulting in the accumulation of p53 and its phosphorylation [33]. The results showed that melatonin upregulated the expression of MT1 and MT2 with the enhanced expression of p53 in the mouse uterus. p53 has been identified as a crucial factor for implantation and is the upstream regulator of the expression of *LIF* [9]. The results indicate that p53 may be a downstream element of MT1/2 activation and melatonin could regulate p53 and then upregulate LIF expression to improve embryo implantation. We also recognize the limitations of our study. Currently, we cannot distinguish which melatonin receptors, MT1, MT2 or both, are required for the signal transduction pathway. MT1 and MT2 or both knockout transgenic animal models will be needed for further studies.

## Conclusion

In conclusion, light pollution jeopardized reproductive outcome, which was indicated by a significantly decreased number of embryo implantation sites in the uteri of mice that were maintained under long photoperiodic exposure. This observation has significant clinical implications for pregnant night shift workers such as nurses and other women of reproductive age who work at night. We also observed that melatonin treatment in pregnant mice had a beneficial effect, including an increase in litter size and litter weight. This was achieved by an increase in the number of embryo implantation sites at the early gestation. A possible molecular mechanism is that melatonin administration increases the E_2_ levels during pregnancy and upregulates p53 expression, which is mediated by the activation of MT1/2 in the uteri of mice. All of the changes improve the microenvironment of the uterus and, thus, the outcomes of pregnancy. If the results of the animal study can be translated to humans, pregnant night shift workers may benefit from melatonin supplementation. However, more animal and clinical studies are required.

## Abbreviations

E_2_: 17β-estradiol
MT: Melatonin
MT1: Melatonin receptor type 1
MT2: Melatonin receptor type 2
P_4_: Progesterone
